# Left Atrioventricular Coupling Index Predicts Poor Prognosis in Acute Myocardial Infarction: A Single-Center Cohort Study

**DOI:** 10.3390/jcdd13020090

**Published:** 2026-02-11

**Authors:** Chuyun Chen, Haolei Huang, Jia Jia, Fangfang Fan, Jie Jiang, Ying Yang, Yan Zhang

**Affiliations:** 1 Department of Cardiology, Peking University First Hospital, Beijing 100034, China; 2211110291@alumni.pku.edu.cn (C.C.); 2411210200@stu.pku.edu.cn (H.H.); jiajia9985@163.com (J.J.); fang9020@126.com (F.F.); jiangjie417@vip.com (J.J.); 2 Institute of Cardiovascular Disease, Peking University First Hospital, Beijing 100034, China; 3 Hypertension Precision Diagnosis and Treatment Research Center, Peking University First Hospital, Beijing 100034, China; 4 State Key Laboratory of Vascular Homeostasis and Remodeling, Peking University, Beijing 100034, China; 5 NHC Key Laboratory of Cardiovascular Molecular Biology and Regulatory Peptides, Peking University, Beijing 100034, China

**Keywords:** left atrioventricular coupling index, echocardiography, major adverse cardiovascular events, cohort study

## Abstract

(1) Background: The left atrioventricular coupling index (LACI) is a novel parameter for evaluating cardiac function. This study focused on its association with major adverse cardiovascular events (MACEs) in acute myocardial infarction (AMI) patients. (2) Methods: A retrospective cohort of AMI patients from Peking University First Hospital was enrolled. All underwent transthoracic echocardiography on admission for LACI measurement. The primary endpoint was MACE (a composite of nonfatal stroke, nonfatal myocardial infarction, and cardiovascular death). (3) Results: Among 843 AMI patients (62.07 ± 12.24 years, 77.94% male), the median LACI was 0.24 (IQR 0.18–0.33). During a median follow-up of 4.31 years, 151 patients (17.91%) developed MACE. The optimal LACI cutoff for risk stratification was 0.257. After multivariable adjustment, each standard deviation increase in LACI was associated with significantly elevated risks of MACE (HR 1.17, 95% CI 1.02–1.34), all-cause death (HR 1.19, 95% CI 1.05–1.35), cardiovascular death (HR 1.33, 95% CI 1.10–1.61), and stroke (HR 1.23, 95% CI 1.05–1.43). (4) Conclusions: LACI is an independent predictor of poor prognosis in AMI patients and may serve as a valuable tool for risk stratification in secondary prevention.

## 1. Introduction

AMI is still one of the most serious public health challenges in China. It is estimated that by 2030, the number of AMI patients in China will reach 23 million, and a considerable number of them are at risk of recurrent cardiovascular adverse events [1]. In the context of the increasing burden of disease, noninvasive tools that can accurately identify and classify risks are crucial to improve the long-term prognosis of patients with AMI.

Currently, the left ventricular ejection fraction (LVEF), widely relied upon in clinical practice, still possesses certain limitations. A large-scale study focusing on patients with stable heart failure revealed that LVEF and mortality showed a nearly linear negative correlation only in the group with reduced ejection fraction (LVEF ≤ 45%); in the group with preserved ejection fraction (LVEF > 45%), even as LVEF continued to increase, it did not lead to a further reduction in mortality risk [2]. Therefore, there is an urgent clinical need for novel indicators that can more sensitively reflect the overall functional state of the heart. In recent years, the focus of cardiac imaging research has shifted from isolated assessment of individual chambers to examining the complex interactions between the atria and ventricles, known as “atrioventricular coupling.” Against this backdrop, LACI has emerged.

LACI, defined as the ratio of left atrial minimum volume (LAVmin) to left ventricular end-diastolic volume (LVEDV), reflects the structural and functional balance between the atrium and ventricle and has become an innovative index to quantify this interaction. An increase in LACI indicates that the expansion of LA is not proportional to that of the LV, suggesting that late adaptive remodeling and coupling are impaired. It can be quantified by using advanced imaging techniques such as cardiac magnetic resonance (CMR), which has recently shown the prognostic significance of LACI in an AMI cohort [3]. However, the use of more accessible, cost-effective and widely available echocardiographic methods for evaluation provides greater potential for rapid clinical integration and dynamic risk stratification.

Currently, the prognostic significance of the echocardiographically derived LACI specifically in AMI patients, particularly in the Chinese population, remains insufficiently explored. Therefore, the primary objective of this study was to comprehensively investigate the association between echocardiographic LACI and the long-term risk of MACE in a large, contemporary cohort of Chinese patients with AMI.

## 2. Materials and Methods

### 2.1. Study Population

From January 2010 to December 2019, patients admitted for AMI in the Department of Cardiology of Peking University First Hospital were recruited. The inclusion criteria were: (1) age ≥ 18 years; (2) diagnosed with AMI [4]; (3) with complete clinical data in the electronic medical record; and (4) echocardiographic images stored on admission that allowed perfect measurement of LA minimal volume (LAVmin; measured at the end of LV diastole) and LV end-diastolic volume (LVEDV). The exclusion criteria were as follows: (1) less than 18 years; (2) with incomplete clinical data; (3) angina; (4) with unsatisfactory echocardiographic images. The study protocol was approved by the Peking University First Hospital ethics committee and conducted in accordance with the Declaration of Helsinki.

Demographic and clinic characteristics of all patients included age, sex, smoking, alcohol use, disease (i.e., hypertension, dyslipidemia, atrial fibrillation, diabetes, cerebrovascular disease, chronic kidney disease), type of myocardial infarction, percutaneous coronary intervention (PCI), and use of aspirin, clopidogrel or ticagrelor, beta-blockers, angiotensin-converting enzyme inhibitors/angiotensin receptor antagonists (ACEI/ARB) and statin. Furthermore, laboratory measurements included total cholesterol, low-density lipoprotein cholesterol (LDL-C), high-density lipoprotein cholesterol (HDL-C), triglycerides, creatinine, BNP, and CK-MB. All the information above was extracted from the electronic health records.

### 2.2. Echocardiography

Echocardiography was measured in all AMI patients on admission using an ultrasound system (Vivid-7; General Electric, Boston, MA, USA) with a 3 MHz transducer and digitally stored. For the patients who underwent PCI, echocardiography was conducted prior to the procedure to ensure that volumetric measurements reflected acute-phase baseline cardiac structure without hemodynamic perturbations induced by revascularization. Images were digitally stored for offline analysis. The volume of the left atrium and left ventricle, measured using the biplane Simpson’s method of disks in accordance with current ASE/EACVI recommendations [5], was measured and recorded by two experienced physicians who were blinded to patient baseline characteristics, treatment allocation, and clinical outcomes. LAEF was defined as [(LAVmax − LAVmin)/LAVmax] × 100. LACI was defined as the ratio between LAVmin and LVEDV [6]. LA and LV volumes were measured in the same end-diastolic phase defined by the mitral valve closure. LACI reflected greater impairment of left atrioventricular coupling, with higher values indicating greater disproportion between LA and LV volumes at ventricular end-diastole. All measurements were made over three consecutive cardiac cycles, and average values were used for the final analyses.

### 2.3. Outcomes

The primary endpoint was MACE (defined as a composite of nonfatal stroke, nonfatal myocardial infarction, and cardiovascular death). The secondary endpoints included stroke, myocardial infarction, all-cause death, and cardiovascular death. The outcome data originated from the Chinese Center for Disease Control and Prevention and the Beijing Municipal Health Commission. Information was also recorded by telephone interviews with patients or their family members, performed by trained reviewers who were blinded to the echocardiography measurements. All the patients were followed up (with no missing) after initial echocardiography until 31 December 2021, or the occurrence of the endpoint of this study.

### 2.4. Statistical Analysis

The variables are expressed as the mean ± standard deviation, median (interquartile range), or number (percentage). Student’s *t*-test, chi-squared test, or Fisher’s exact test were used appropriately to make comparisons among the groups. An optimal dichotomization cutoff value for LACI was determined using Youden’s Index. The association between LACI and MACE was modeled using univariable and multivariable Cox proportional risk regression models, with hazard ratio (HR) and 95% confidence interval (CI). Factors with a *p*-value of <0.1 in univariate Cox regression and those that were clinically considered meaningful were selected for further analysis in multivariate Cox proportional hazard models. We used restricted cubic splines with four knots to flexibly model the association between LACI and the outcomes. The mean LACI value (0.28) was selected as the reference point. For each LACI value, the hazard ratio (HR) was calculated by exponentiating the difference in the linear predictor (log HR) between that LACI value and the reference value, derived from the fitted Cox model. The 95% confidence intervals (CIs) were computed based on the standard errors of the linear predictors. The HRs were plotted as a function of LACI, with the 95% CIs represented as a shaded ribbon. A horizontal dashed line at HR = 1 was included to indicate the threshold of no differential risk relative to the reference LACI value. All statistical analyses were performed using R software (version 4.3.3, http://www.R-project.org, accessed on 13 January 2026) and Empower (http://www.empowerstats.com, X&Y Solutions, Inc., Boston, MA, USA, accessed on 13 January 2026). A two-tailed *p*-value of <0.05 was considered statistically significant.

## 3. Results

### 3.1. Baseline Characteristics

As shown in Table 1, a total of 843 AMI patients were included. The mean age was 62.07 ± 12.24 years, and 657 (77.94%) were male. Overall, 461 patients (54.69%) were STEMI, and 382 patients (45.31%) were NSTEMI. The median LACI value was 0.24 (interquartile range, 0.18–0.33). Patients who experienced MACE were significantly older (65.48 ± 11.61 vs. 61.32 ± 12.25, *p* < 0.001) and showed increased prevalence of hypertension (73.33% vs. 63.19%, *p* = 0.018), atrial fibrillation (11.33% vs. 5.80%, *p* = 0.015), diabetes (60.67% vs. 35.65%, *p* < 0.001), cerebrovascular disease (28.00% vs. 14.64%, *p* < 0.001) and chronic kidney disease (19.33% vs. 8.42%, *p* < 0.001) compared with patients without MACE (Table 1). Regarding echocardiographic characteristics, patients who experienced MACE showed significantly greater values of LACI (0.27 (0.21–0.37) vs. 0.23 (0.18–0.32), *p* < 0.001) (Table 2).

### 3.2. Clinical Outcomes

During a median follow-up period of 4.31 years (interquartile range, 3.02–5.76 years), 151 (17.91%) cases of MACE occurred, 82 (9.73%) had nonfatal stroke, 111 (13.17%) had recurrent myocardial infarction, 126 (14.95%) were identified as all-cause death, and 47 (5.58%) were identified as cardiovascular death.

**Figure 1:**
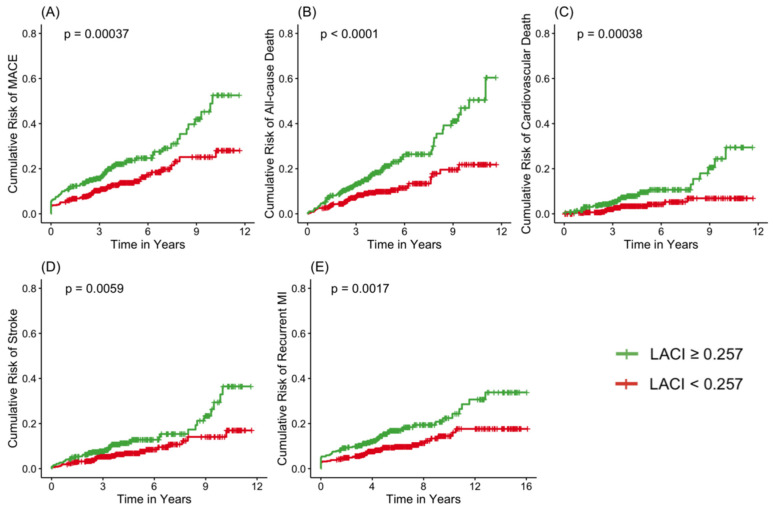
The cumulative risk curve of LACI for MACE (**A**), All-cause Death (**B**), Cardiovascular Death (**C**), Stroke (**D**) and recurrent myocardial infarction. (**E**) [green: LACI ≥ 0.257; red: LACI ≤ 0.257].

In the overall cohort, the Youden Index identified an optimal LACI cutoff of 0.257 to classify patients into low- and high-risk groups (*p* = 0.00012). This value effectively stratified patient risk, with those above the threshold exhibiting a significantly poorer prognosis. Kaplan–Meier survival analysis demonstrated that patients with LACI ≥ 0.257 had significantly lower event-free survival rates for MACE, all-cause death, cardiovascular death, and stroke (all log-rank *p* < 0.01), visually confirming the robust discriminative ability of this cutoff value in risk stratification (Figure 1).

### 3.3. Association Between LACI and Outcomes

**Figure 2:**
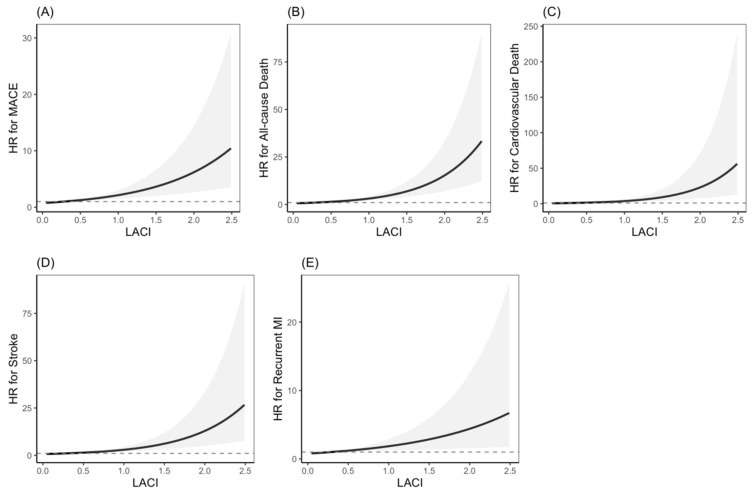
The spline smoothing curve of hazard ratio (HR) of LACI for MACE (**A**), All-cause Death (**B**), Cardiovascular Death (**C**), Stroke (**D**) and recurrent myocardial infarction (**E**).

The restricted cubic spline curves (Figure 2) illustrated a near-linear dose–response relationship between LACI and the risks of MACE, all-cause death, cardiovascular death, and stroke. Notably, the risk increased progressively with higher LACI values, without evidence of a threshold effect, suggesting that LACI behaves as a continuous risk marker. In contrast, the association between LACI and recurrent myocardial infarction was weaker and non-significant, implying that the pathophysiological pathways linking LACI to prognosis may differ from those primarily driving recurrent coronary events.

After multivariable adjustment for age, sex, hypertension, diabetes, cerebrovascular disease, chronic kidney disease, atrial fibrillation, type of myocardial infarction, percutaneous coronary intervention, LDL-C, triglyceride, LVEF, and secondary prevention drugs for coronary heart disease, LACI remained independently associated with an increased risk of adverse outcomes. Specifically, per 1-SD increase in LACI was associated with a 17% higher risk of MACE (HR 1.17, 95% CI 1.02–1.34), a 19% higher risk of all-cause death (HR 1.19, 95% CI 1.05–1.35), a 33% higher risk of cardiovascular death (HR 1.33, 95% CI 1.10–1.61), and a 23% higher risk of stroke (HR 1.23, 95% CI 1.05–1.43) (Table 3).

## 4. Discussion

### 4.1. Principal Findings

Through mid- to long-term follow-up of 843 patients with acute myocardial infarction (AMI), this study provides a systematic evaluation in a Chinese population of the prognostic value of the LACI measured by transthoracic echocardiography. The primary finding is that LACI serves as an independent predictor of MACE, all-cause death, cardiovascular death, and non-fatal stroke following AMI. The results of this study not only confirmed the important role of LACI in risk stratification of AMI but also provided direct evidence for the clinical application of this novel and noninvasive parameter in the Chinese population. At present, LACI has shown important application in risk stratification and prognosis assessment of a series of cardiovascular diseases, such as heart failure (especially heart failure with preserved ejection fraction), atrial fibrillation, myocardial infarction and cardiomyopathy [7,8,9].

### 4.2. Pathophysiological Basis of LACI and Its Relationship with AMI

The LACI derives its prognostic power from its deep reflection of the post-AMI cardiac compensation and decompensation process. The pathophysiology begins with impaired left ventricular diastolic function, which elevates left ventricular filling pressure. This, in turn, imposes a volume overload on the left atrium, triggering acute compensatory dilation to maintain cardiac output. Consequently, the minimum left atrial volume increases, serving as a sensitive surrogate for elevated filling pressure and leading to a rise in LACI [7,9]. However, persistent overload can lead to failure of the Frank–Starling mechanism and a decline in left atrial systolic function [10].

Although this atrial dilation may disappear with the recovery of myocardial stunning in some patients, it usually persists in patients with larger infarct size or older age, which means irreversible atrial remodeling [11]. In this case, LACI, as a comprehensive parameter, skillfully captures the compensation mechanism of pathological enlargement of the left atrium, which leads to the impairment of left ventricular function–atrioventricular decoupling state [12]. Compared with evaluating single left atrial or left ventricular parameters alone, LACI reveals the compensatory load of the heart on AMI injury earlier and comprehensively from the perspective of ventricular interaction, which may form the basis of its effective prognosis.

### 4.3. Independent Prognostic Value and Endpoint Specificity of LACI

Although there was no significant difference in CK-MB (a marker of acute myocardial injury) between the two groups in this study, BNP, which reflects ventricular wall stress and chronic remodeling, was significantly elevated in the MACE group. This comparison indicates that long-term cardiovascular risk is mainly caused by sustained post-infarction cardiac remodeling and dysfunction, rather than just the initial infarct size. In addition, this study observed that patients with MACE had lower baseline lipid levels, which may be consistent with the “lipid paradox” phenomenon, where the lowest LDL-C quartiles are typically associated with the highest mortality rate. This association may be mediated by comorbidities, excessive use of statins, and impaired nutritional status [13]. Importantly, even after extensive adjustment of confounding factors (including age, complications, revascularization strategies, LVEF, and secondary preventive drugs), LACI still shows strong prognostic value (Table 3). This indicates that the prognostic information captured by LACI, reflecting the decoupling status of the atria and ventricles, exceeds traditional risk markers and disease severity indices.

Notably, the prognostic information conveyed by LACI showed distinct endpoint specificity. It showed the strongest association with cardiovascular death (adjusted HR 1.33 per 1-SD, Table 3). Conversely, multivariable analysis and dose–response curves consistently indicated a weaker, statistically nonsignificant association between LACI and recurrent myocardial infarction (Table 3). This finding suggests that it is more closely associated with endpoint events directly related to heart pump dysfunction and electromechanical instability. In contrast, recurrent myocardial infarction is primarily driven by instability within the coronary artery system, including plaque rupture, thrombosis, vasospasm, and inflammation [14], rather than direct impairment of pump function or atrioventricular coupling. Therefore, although LACI is a robust indicator of cardiac functional and structural remodeling, its value in predicting the occurrence or recurrence of coronary events themselves may be limited, consistent with the findings of Lange et al., who reported that LACI was not associated with reinfarction (HR 2.9 [95% CI 0.38–21.2], *p* = 0.31) [3]. This suggests that in future comprehensive risk assessments for patients with AMI, LACI may have complementary value to indicators such as coronary imaging or inflammatory biomarkers.

Our study used a restricted cubic spline curve to clarify the precise dose–response relationship between LACI and end-point events (Figure 2). We observed that MACE, the risk of all-cause death, cardiovascular death and stroke increased linearly with the increase in LACI, especially when the LACI value was high. This continuous relationship has no obvious threshold effect, indicating that the incremental increase of LACI may bring higher and higher risks, even within the traditionally defined “normal” range, thus supporting the treatment of LACI as a continuous risk marker rather than just a dichotomy tool.

### 4.4. Optimal Cutoff Value and Risk Stratification

In our study, the optimal LACI cutoff value, measured by transthoracic echocardiography, was determined as 0.257 using the Youden index, which effectively distinguished long-term MACE risk in patients with AMI. Survival analysis showed that the high-risk group defined by this cutoff had significantly lower event-free survival throughout mid- to long-term follow-up (Figure 1), indicating that LACI-based risk stratification has sustained clinical significance.

It is noteworthy that the optimal echocardiographic LACI cutoff value identified in our study (0.257) aligns closely with the findings of a recent cardiac CT study by Pezel et al. [15], but was lower than values reported in some CMR studies (e.g., 34.7% reported by Lange et al. [3]). The observed discrepancies likely arise from several key methodological divergences: firstly, in statistical modeling—while both studies treat LACI as a continuous variable, we report hazard ratios (HRs) per 1 standard deviation (SD) increase, whereas Lange et al. [3] present HR per 1% absolute increase; secondly, in endpoint definition—our MACE composite includes non-fatal stroke, while theirs incorporates heart failure hospitalization; and thirdly, in imaging modality—CMR, as the volumetric gold standard, offers superior spatial resolution and reproducibility for detecting subtle pathophysiological LACI changes, whereas echocardiography, though more accessible, exhibits greater measurement variability due to acoustic window limitations and operator dependency. Crucially, despite these technical variations, the core conclusions remain highly consistent. All three imaging techniques converge in demonstrating that the left atrioventricular decoupling state captured by LACI constitutes a fundamental pathophysiological mechanism driving adverse cardiovascular outcomes. This consensus robustly suggests that LACI ≥ 25% may serve as a universal, modality-agnostic marker of elevated risk, transcending specific measurement methodologies.

### 4.5. Clinical Application Challenges and Future Prospects

However, the clinical application of this indicator is facing challenges, mainly due to the lack of a standardized reference range. This limitation stems from the differences in measurement methods, including different imaging methods, such as echocardiography, cardiac magnetic resonance (CMR), and computed tomography, as well as the heterogeneity within the study population affected by factors such as age, gender, and ethnicity [8,9].

The paramount clinical implication of this study is that powerful prognostic information can be derived from LACI obtained via accessible, cost-effective echocardiography. In clinical practice, LACI can serve as a potential supplement to traditional risk stratification tools (e.g., GRACE score) and LVEF [16]. It is particularly useful for identifying the “hidden” high-risk patients with preserved LVEF but significant atrioventricular decoupling, who face an elevated risk of heart failure and cardiac death [12,17,18]. Future research should focus on prospectively validating the echocardiographic LACI cutoff values in multicenter cohorts, exploring the prognostic significance of dynamic changes in LACI, and initiating interventional studies to determine whether therapies specifically aimed at reducing LACI can ultimately improve the long-term outcomes of AMI patients.

## 5. Limitations

As a single-center retrospective study, potential information bias and limited generalizability may affect its applicability to other regions or hospital settings. Although the relationship between LACI and events was explored for non-linearity using restricted cubic splines, this study primarily reports risk per standard deviation increase in LACI; future studies could further explore its optimal cutoff values in different subgroups. LACI has not yet been integrated into the GRACE score or other routine clinical risk assessment tools, and further research is still needed to validate its incremental value beyond traditional risk stratification systems. Finally, although LACI demonstrates a strong prognostic correlation, whether interventions targeting LACI can improve outcomes requires confirmation through prospective investigations.

## 6. Conclusions

This study establishes echocardiography-derived LACI as an independent predictor of adverse long-term outcomes in AMI patients. Each standard deviation increase in LACI was associated with elevated risks of major cardiovascular events, all-cause death, and stroke. A clinically applicable cutoff of 0.257 effectively identified high-risk patients. These findings support LACI as a practical, incremental risk stratification tool. Future research should focus on multicenter validation, measurement standardization, and interventional studies to confirm clinical utility.

## Figures and Tables

**Table 1:** Baseline clinical characteristics of all the AMI patients.

Variables	Total (*n* = 843)	Non-MACE Group (*n* = 692)	MACE Group (*n* = 151)	*p*
age, mean ± SD, years	62.07 ± 12.24	61.32 ± 12.25	65.48 ± 11.61	<0.001
male, *n* (%)	657 (77.94)	541 (78.18)	116 (76.82)	0.715
smoking, *n* (%)	501 (59.79)	412 (59.88)	89 (59.33)	0.901
alcohol use, *n* (%)	273 (32.58)	227 (32.99)	46 (30.67)	0.582
hypertension, *n* (%)	546 (65.00)	436 (63.19)	110 (73.33)	0.018
dyslipidemia, *n* (%)	524 (62.38)	434 (62.90)	90 (60.00)	0.507
atrial fibrillation, *n* (%)	57 (6.79)	40 (5.80)	17 (11.33)	0.015
diabetes, *n* (%)	337 (40.12)	246 (35.65)	91 (60.67)	<0.001
cerebrovascular disease, *n* (%)	143 (17.02)	101 (14.64)	42 (28.00)	<0.001
chronic kidney disease, *n* (%)	87 (10.37)	58 (8.42)	29 (19.33)	<0.001
type of AMI, *n* (%)				0.023
STEMI	461 (54.69)	391 (56.50)	70 (46.36)	
NSTEMI	382 (45.31)	301 (43.50)	81 (53.64)	
PCI (*n*, %)	659 (78.17)	551 (79.62)	108 (71.52)	0.029
BNP (pg/mL), median (IQR)	199.00 (58.90–502.00)	164.95 (52.00–407.00)	469.10 (174.00–1074.00)	<0.001
CK-MB (U/L), median (IQR)	20.00 (3.30–119.24)	23.90 (3.10–124.80)	13.35 (3.40–85.64)	0.564
creatinine (μmol/L), median (IQR)	85.00 (73.85–100.28)	83.67 (73.70–97.00)	90.00 (74.90–119.61)	<0.001
total cholesterol (mmol/L), mean ± SD	4.30 ±1.22	4.37 ± 1.24	3.96 ± 1.06	<0.001
triglyceride (mmol/L), median (IQR)	1.42 (1.02–2.04)	1.45 (1.03–2.08)	1.30 (0.98–1.78)	0.019
HDL-C (mmol/L), mean ± SD	0.94 ± 0.22	0.94 ± 0.22	0.91 ± 0.25	0.027
LDL-C (mmol/L), mean ± SD	2.51 (2.00–3.15)	2.57 (2.01–3.23)	2.36 (1.94–2.83)	<0.001
aspirin, *n* (%)	775 (93.94)	637 (94.37)	138 (92.00)	0.271
ticagrelor, *n* (%)	181 (21.94)	171 (25.33)	10 (6.67)	<0.001
clopidogrel, *n* (%)	565 (68.48)	444 (65.78)	121 (80.67)	<0.001
beta-blockers, *n* (%)	689 (83.52)	568 (84.15)	121 (80.67)	0.299
ACEI/ARB, *n* (%)	552 (66.91)	457 (67.70)	95 (63.33)	0.304
statin, *n* (%)	760 (92.12)	626 (92.74)	134 (89.33)	0.161

Abbreviations: AMI, acute myocardial infarction; MACE, major adverse cardiovascular event; NSTEMI, non-ST-segment elevation myocardial infarction; STEMI, ST-segment elevation myocardial infarction; PCI, percutaneous coronary intervention; HDL-C, high-density lipoprotein cholesterol; LDL-C, low-density lipoprotein cholesterol; ACEI/ARB, angiotensin-converting enzyme inhibitor/angiotensin receptor antagonists.

**Table 2:** Echocardiographic characteristics of all the AMI patients.

Variables	Total (*n* = 843)	Non-MACE Group (*n* = 692)	MACE Group (*n* = 151)	*p*
LVEF	54.89 ± 10.93	55.49 ± 10.64	52.11 ± 11.81	<0.001
LVESV	37.00 (26.20–52.00)	36.00 (26.00–50.00)	41.00 (27.45–65.50)	0.006
LVEDV	86.00 (67.20–108.00)	85.05 (67.00–105.70)	89.50 (68.10–116.60)	0.115
LAEF	57.51 ± 11.00	58.13 ± 10.55	54.66 ± 12.51	<0.001
LAVmin	20.00 (15.10–28.30)	19.40 (14.67–27.00)	24.20 (17.40–36.15)	<0.001
LAVmax	51.40 (41.10–63.65)	50.00 (40.40–62.30)	57.70 (45.55–76.60)	<0.001
LACI	0.24 (0.18–0.33)	0.23 (0.18–0.32)	0.27 (0.21–0.37)	<0.001

**Table 3:** The association between LACI and MACE.

	Events,	Crude		Model I		Model II	
	*n* (%)	HR (95%CI)	*p*	HR (95%CI)	*p*	HR (95%CI)	*p*
MACE	151 (17.91%)						
LACI (per 1-SD increase)		1.23 (1.12, 1.36)	<0.001	1.17 (1.05, 1.30)	0.005	1.17 (1.02, 1.34)	0.024
All-cause death	126(14.95%)						
LACI (per 1-SD increase)		1.37 (1.25, 1.50)	<0.001	1.21 (1.09, 1.35)	<0.001	1.19 (1.05, 1.35)	0.007
Cardiovascular death	47(5.58%)						
LACI (per 1-SD increase)		1.44 (1.26, 1.64)	<0.001	1.31 (1.13, 1.52)	<0.001	1.33 (1.10, 1.61)	0.003
Stroke	82(9.73%)						
LACI (per 1-SD increase)		1.34 (1.20, 1.50)	<0.001	1.26 (1.11, 1.42)	<0.001	1.23 (1.05, 1.43)	0.009
Recurrent MI	111(13.17%)						
LACI (per 1-SD increase)		1.19 (1.05, 1.34)	0.006	1.11 (0.97, 1.27)	0.135	1.12 (0.95, 1.32)	0.173

Model I has been adjusted for age and sex. Model II includes the factors from Model I with further adjustment for hypertension, diabetes, cerebrovascular disease, chronic kidney disease, atrial fibrillation, type of myocardial infarction, percutaneous coronary intervention, LDL-C, triglyceride, left ventricular ejection fraction and secondary prevention drugs for coronary heart disease.

## Data Availability

The data that support the findings of this study are available from the corresponding author upon reasonable request after the request is submitted and formally reviewed and approved by the ethics committee of Peking University First Hospital. Requests to access the datasets should be directed to drzhy1108@163.com.

## Abbreviations

The following abbreviations are used in this manuscript:

LACI	The left atrioventricular coupling index
MACE	Major adverse cardiovascular events
AMI	Acute myocardial infarction
LVEF	Left ventricular ejection fraction
LAVmin	LA minimal volume
LVEDV	Left ventricular end-diastolic volume
CMR	Cardiac magnetic resonance
PCI	Percutaneous coronary intervention
ACEI/ARB	Angiotensin-converting enzyme inhibitor/angiotensin receptor antagonist
LDL-C	Low-density lipoprotein cholesterol
HDL-C	High-density lipoprotein cholesterol
HR	Hazard ratio
CI	Confidence interval
CIs	Confidence intervals
CCTA	Coronary CT angiography
